# The OX40-OX40L Co-Stimulatory Pathway as a Shared Driver of Immune Persistence in Skin and Airway Inflammation

**DOI:** 10.1007/s12016-026-09187-x

**Published:** 2026-07-24

**Authors:** Francisco José Navarro-Triviño, Tiago Torres, Ricardo Ruiz-Villaverde, José Luis Moreno-Amador, Pedro Mendes-Bastos

**Affiliations:** 1 Universidad de Granada, Granada, Spain; 2 Servicio de Dermatología, Hospital Universitario San Cecilio, Granada, Spain; 3 Instituto Biosanitario de Granada (ibs.GRANADA), Granada, España; 4 Serviço de Dermatologia, Centro Hospitalar Universitário do Porto, Instituto de Ciências Biomédicas Abel Salazar. Universidade do Porto, Porto, Portugal; 5 Sanofi, Madrid, Spain; 6 Serviço de Dermatologia, Hospital CUF Descobertas, Lisboa, Portugal

**Keywords:** OX40, OX40L, Type 2 inflammation, Immune memory, Barrier tissues, Atopic dermatitis, Airway inflammation

## Abstract

The OX40-OX40L co-stimulatory pathway has emerged as an important regulator of T-cell persistence and immune memory. While traditionally associated with type 2 inflammation, its role extends across multiple barrier tissues, including the skin and respiratory mucosa, where it integrates epithelial signals with adaptive immune responses. To provide a comprehensive and integrative overview of the OX40-OX40L axis as a shared driver of immune persistence across skin and airway inflammatory diseases and to discuss its therapeutic implications. A narrative review was conducted based on a focused literature search of PubMed, Scopus, and Web of Science up to February 2026. Experimental, translational, and clinical studies addressing OX40-OX40L biology and therapeutic targeting were included. OX40 signaling acts as a late co-stimulatory pathway that sustains effector T-cell survival, promotes tissue-resident memory T-cell formation, and modulates regulatory T-cell function. In barrier tissues, epithelial-derived alarmins such as TSLP and IL-33 induce OX40L expression on antigen-presenting cells, reinforcing type 2-polarized immune responses. This axis contributes to chronic inflammation in atopic dermatitis, asthma, allergic rhinitis, and nasal polyposis, supporting the concept of shared mechanisms of immune persistence across type 2 inflammatory diseases. Beyond type 2 immunity, OX40-OX40L signaling also participates in Th1-, Th17-, and Th22-mediated responses, as well as in autoimmune, fibrosing, and lymphoproliferative disorders. Therapeutic targeting of this pathway, particularly with monoclonal antibodies such as amlitelimab, has demonstrated clinically meaningful efficacy and suggests potential for durable disease modification. The OX40-OX40L axis represents an important immunological pathway linking epithelial activation to adaptive immune memory across barrier tissues. Its role in sustaining immune persistence provides a conceptual framework for understanding chronic inflammatory diseases and supports continued investigation of therapies targeting upstream co-stimulatory pathways.

## Introduction

OX40 (CD134) is a costimulatory receptor of the tumor necrosis factor receptor (TNFR) superfamily whose expression is induced upon T-cell activation. It is primarily expressed on activated CD4⁺ T cells, but can also be detected on CD8⁺ T cells, memory T cells, and selected regulatory T-cell subsets [1]. Its ligand, OX40L (CD252/TNFSF4), is mainly expressed on antigen-presenting cells (APCs), particularly dendritic cells, although expression has also been described on B cells, endothelial cells, mast cells, and basophils under inflammatory conditions [2]. Engagement of OX40 by OX40L enhances T-cell expansion, survival, and differentiation into effector and memory populations, while reducing suppressive regulatory T-cell function, thereby consolidating adaptive immune responses beyond initial antigen recognition [3, 4].

Unlike early costimulatory signals, the OX40-OX40L axis acts as a late co-stimulatory pathway that reinforces T-cell persistence and immunological memory [5, 6]. OX40L signaling also modulates APC function and cytokine production, and its expression on dendritic cells is essential for effective sensitization and memory formation, particularly in cutaneous immune responses [7].

Epithelial-derived alarmins regulate OX40-OX40L signaling in barrier tissues such as the skin and respiratory mucosa by inducing OX40L expression on dendritic cells, thereby favoring the expansion and persistence of type 2-polarized OX40⁺ T cell [8–10]. This axis contributes to the chronic T-cell-mediated inflammation characteristic of atopic dermatitis and related type 2 disorders.

Consistent with this biology, increased expression of both OX40 and OX40L has been demonstrated in lesional skin and peripheral blood of patients with AD, where it correlates with disease activity and chronicity [11–13]. Similar mechanisms operate in the airways [14], where OX40-OX40L signaling contributes to allergen-driven Th2 inflammation, with basophils acting as non-conventional APCs capable of initiating and sustaining OX40-dependent immune responses [15, 16].

These findings position the OX40-OX40L axis as a central amplifier of immune persistence in barrier tissues, integrating epithelial danger signals with APCs and T lymphocytes to promote long-lived effector and memory T-cell responses, limit regulatory control, and facilitate chronic inflammation. Increasing evidence suggests that OX40 signaling extends beyond classical Th2 polarization, supporting immune plasticity across multiple T-cell lineages during chronic disease states [17]. These features make the OX40-OX40L axis a compelling therapeutic target and provide the rationale for the growing clinical interest in its modulation across type 2 inflammatory disorders.

## Materials and Methods

### Study Design

This narrative and integrative review synthesizes experimental, translational, and clinical evidence on the OX40-OX40L costimulatory axis in barrier tissues of type 2 inflammatory disease. This review focuses on the role of OX40 and OX40L in the skin and airways, their impact on effectors, memory, and regulatory T-cell programs, and the translational development of monoclonal antibodies targeting this pathway.

### Literature Search and Selection

A focused literature search was conducted in PubMed/MEDLINE, Scopus, and Web of Science, and updated through February 2026. Search terms included combinations of “OX40”, “OX40L”, “TNFRSF4”, “TNFSF4”, “co-stimulation”, “type 2 inflammation”, and disease-specific terms such as atopic dermatitis, asthma, allergic rhinitis, nasal polyposis, contact dermatitis, chronic urticaria, vitiligo, and fibrosing skin disorders.

Articles were screened by title and abstract, followed by full-text review when relevant. The selection was based on the relevance of the immunological mechanisms or therapeutic targeting of the OX40-OX40L pathway. We included experimental studies, translational research, clinical trials, and relevant peer-reviewed review articles. This review provides a qualitative and integrative synthesis rather than a systematic or quantitative analysis.

### Signaling Mechanisms and Immunological Functions of the OX40-OX40L Axis

OX40 (TNFRSF4) is a member of the tumor necrosis factor receptor (TNF) superfamily, and its ligand OX40L (TNFSF4) belongs to the TNF ligand family. Their interaction constitutes a major co-stimulatory pathway that amplifies and sustains T cell-mediated immune responses [18]. Table 1 summarizes the cellular sources and functional roles of OX40 and OX40L across immune and stromal compartments.

**Table 1:** Cellular sources and functions of OX40 and OX40L

Cell type	OX40	OX40L	Functional consequence
CD4⁺ T cells	**↑**	**–**	Survival, TRM, cytokines
CD8⁺ T cells	**↑**	**–**	Memory, cytotoxicity
Regulatory T cells	**↑**	**–**	Suppressed function
Dendritic cells	**–**	**↑**	T-cell priming
Basophils	**–**	**↑**	Th2 initiation
Mast cells	**–**	**↑**	IgE loop, Th2
Endothelial cells	**–**	**↑**	T-cell trafficking
Fibroblasts	**–**	**↑**	Fibrosis

The OX40-OX40L interaction typically occurs 24–72 h after antigen recognition and functions as a late-phase co-stimulatory pathway that prolongs T-cell activation, clonal expansion, and effector cell survival [17]. OX40 signaling is mediated by the recruitment of TNF receptor-associated factor (TRAF) adaptor proteins, primarily TRAF2, TRAF5, and TRAF6, leading to activation of NF-κB, PI3K-AKT, ERK, and STAT5 pathways. These cascades promote T-cell survival and proliferation [19], enhance effector cytokine production in a context-dependent manner [20], and suppress regulatory programs by downregulating FoxP3 and CTLA-4 expression [21], facilitating tissue migration and residency via chemokine receptor induction [22].

OX40 is transiently induced on activated T cells following T-cell receptor engagement and CD28-dependent co-stimulation [23]. In parallel, OX40L is upregulated on activated antigen-presenting cells, particularly dendritic cells, macrophages, and B lymphocytes, and can also be expressed on endothelial cells, mast cells, and basophils under inflammatory conditions [24]. Functional studies established that OX40-OX40L interactions are essential for dendritic cell-dependent T-cell co-stimulation [25], and the absence of OX40L on dendritic cells in murine models of contact hypersensitivity results in defective sensitization and impaired CD4⁺ T-cell activation [26].

Identification of the TSLP-OX40-OX40L axis provided a mechanistic link between epithelial barrier disruption and adaptive Th2 immunity [9]. TSLP released by injured epithelial cells conditions dendritic cells toward a pro-Th2 phenotype characterized by high OX40L expression and IL-12suppression, thereby promoting efficient priming, survival, and reactivation of inflammatory Th2 cells [27]. Consistently, experimental blockade of OX40L reduces Th2 cell reactivation, IgE production, and allergic inflammation in murine models and non-human primates [28], establishing this axis as a central driver of chronic type 2 immune responses.

Pioneering work by Croft and colleagues [3, 4] established OX40 as a hierarchical regulator of late co-stimulatory signaling that sustains T-cell activation, immune-cell metabolism, and long-term immune memory in both CD4⁺ and CD8⁺ T cells [17], and can partially sustain T-cell responses despite inhibitory signaling through CTLA-4 and PD-1 [29, 30].

OX40 signaling operates within a hierarchical and temporally ordered co-stimulatory network that shapes T cell fate and long-term persistence. Initial T-cell priming requires antigen recognition and early CD28-mediated co-stimulation via CD80-CD86, which establishes the activation threshold for clonal expansion and supports memory CD4⁺ [17, 31] and regulatory T-cell maintenance [32], whereas memory CD8⁺ T-cell homeostasis relies predominantly on cytokines such as IL-7 and IL-15 [33, 34]. Only after this priming phase are inducible TNFR family members, including OX40, 4-1BB, and GITR, engaged to sustain effector survival, prolong clonal expansion, and consolidate immunological memory [35, 36], positioning the OX40-OX40L axis as a late-phase co-stimulatory pathway that reinforces T-cell persistence in chronic inflammatory settings.

OX40 is essential for the generation and maintenance of memory CD4⁺ T cells, promoting effector-to-memory differentiation and efficient recall responses in a TRAF2- and NF-κB-dependent manner [37], as evidenced by impaired memory formation and reduced adaptive immunity in OX40-deficient mice [38].

The OX40-OX40L axis regulates immune balance and T-cell plasticity by limiting regulatory T-cell differentiation and suppressing function while promoting the expansion and persistence of effector T-cells. OX40 signaling restrains peripheral Foxp3⁺ Treg induction and attenuates established Treg suppressive capacity through proliferative and metabolic reprogramming [3, 39], while favoring Th2 or Th1 differentiation in IL-4- or IFN-γ-rich environments, respectively [17]. OX40 signaling provides a mechanistic basis for tissue-specific immune deviation and its preferential role in sustaining type 2 skewed inflammation in barrier tissues such as the skin and mucosa through this dual modulation of regulatory and effector compartments [40].

In addition to TSLP, epithelial-derived cytokines, such as interleukin (IL)−33, positively regulate the OX40-OX40L axis by enhancing Th2 polarization and establishing a reinforcing feedback loop [41, 42]. IL-33 potentiates OX40L-dependent dendritic cell-mediated Th2 responses and upregulates the IL-33 receptor ST2 on T cells, thereby sustaining type 2 inflammatory persistence [40].

OX40L activation on antigen-presenting and stromal cells exerts cell-intrinsic effects that collectively shape immune responses and reinforce inflammatory persistence. OX40L enhances co-stimulatory molecule expression and cytokine production in dendritic cells, consolidating T-cell activation [43], and facilitates T-cell adhesion and tissue infiltration in endothelial cells [44]. OX40L signaling amplifies proinflammatory mediator release and IL-4 production in mast cells and basophils, reinforcing Th2 immune responses. Conversely, OX40L engagement by soluble OX40 can limit mast cell activation by inhibiting Fyn-, PI3K-, and RhoA-dependent signaling pathways [45], attenuating systemic anaphylactic responses [46], and suggesting context-dependent regulatory or bidirectional effects in selected inflammatory settings. Collectively, these mechanisms establish a positive feedback loop that sustains T-cell activation, promotes immune memory, and facilitates the transition from acute to chronic inflammation in barrier tissues.

**Fig. 1:**
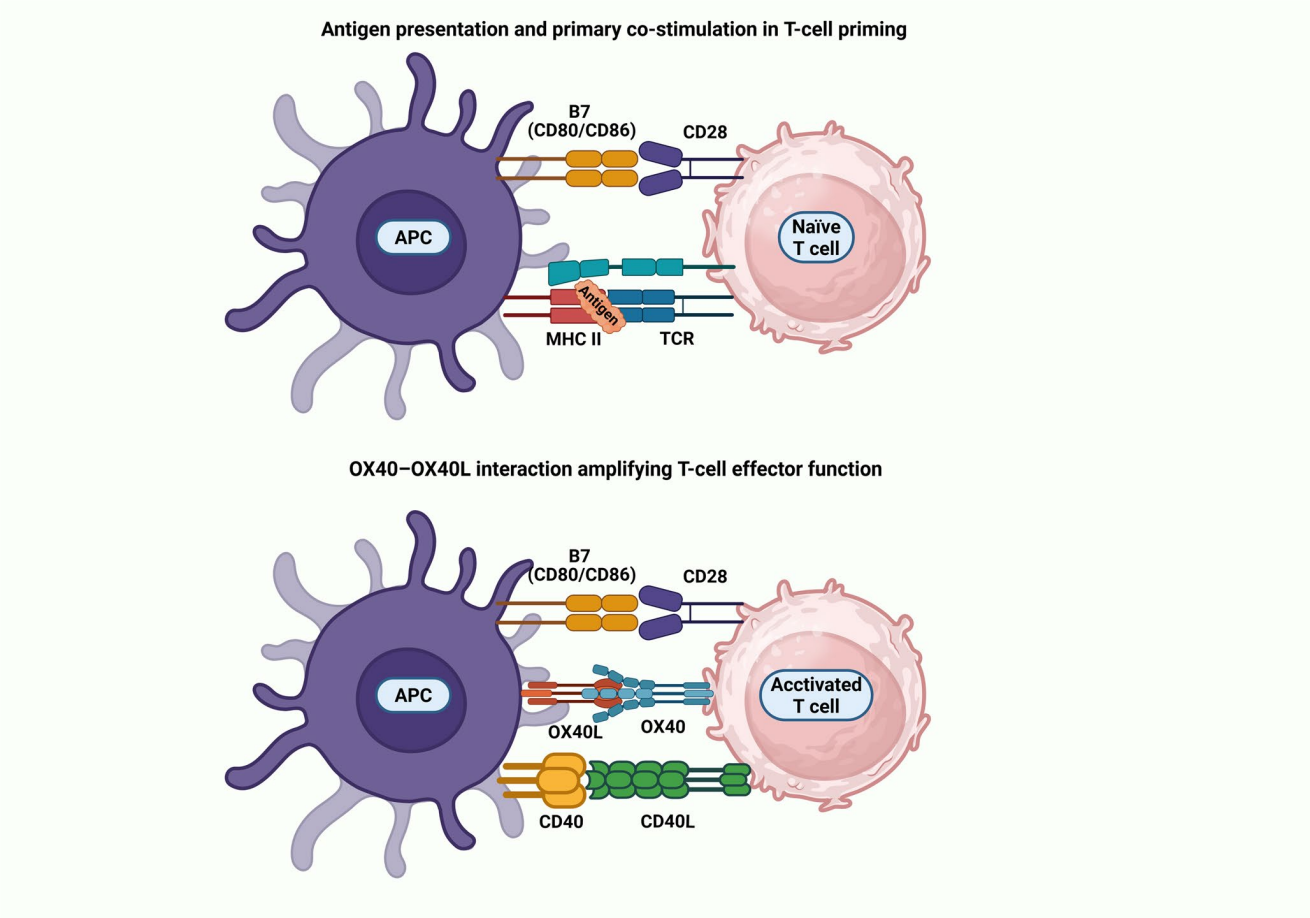
T-cell co-stimulatory signals’ temporal organization during immune activation T-cell priming is initiated by antigen recognition through the TCR-MHC complex and early CD28-CD80/86 co-stimulation. Inducible co-stimulatory pathways, such as OX40-OX40L, operate during the later stages of the immune response, reinforcing effector T cell survival, persistence, and functional commitment. Created with BioRender.com

Beyond classical Th2 polarization, the OX40-OX40L axis acts during the late phase of T-cell responses to support the differentiation, expansion, and long-term persistence of multiple effector subsets, including Th1, Th17, and Th22 cells, particularly in barrier tissues affected by chronic inflammatory conditions [47]. As illustrated in Fig. 1, OX40 expression is induced after CD28-dependent priming and delivers context-dependent co-stimulatory signals that reinforce established immune responses, promoting Th1 differentiation in IL-12-rich environments and Th17/Th22 polarization in settings dominated by IL-6, TGF-β, and epithelial-derived cytokines [48]. OX40 consolidates effector commitment and immunological persistence through these sustained yet temporally restricted effects, positioning this pathway as a central regulator of heterogeneous chronic inflammation at barrier sites.

### OX40-OX40L in Cutaneous and Mucosal Barrier Immunity

The OX40-OX40L axis operates as a key interface between epithelial activation and adaptive immunity in barrier tissue [49]. In the skin, OX40-OX40L signaling contributes to the initiation and maintenance of type 2-mediated inflammation [50], with increased expression of both molecules demonstrated in murine models [51] and lesional skin from patients with atopic dermatitis. Sustained OX40 signaling promotes the persistence of skin-resident memory T cells within clinically non-lesional skin, providing a mechanistic basis for disease recurrence after apparent clinical resolution [52–54]. Co-expression of IL-33 and OX40L in atopic skin correlates with enhanced IL-4, IL-13, and IL-22 production and with greater impairment of epidermal barrier integrity, supporting the role of this axis in chronic epithelial dysfunction [55].

The respiratory mucosa displays a comparable immunological organization. Epithelial-derived signals induce OX40L expression on dendritic cells, basophils, and endothelial cells in the bronchial and nasal epithelium, promoting the expansion of OX40⁺ Th2 cells and driving eosinophilic inflammation, mucus hypersecretion, and airway hyperresponsiveness in asthma [9, 56]. In addition, basophils have been shown in selected experimental models to initiate and amplify key features of type 2 inflammation through OX40L-dependent interactions, even in the absence of classical dendritic cells, and are sufficient to reproduce key features of type 2 inflammation in experimental models [57, 58]. Similar patterns of epithelial alarmin expression, OX40L upregulation, and enrichment of OX40⁺ T cells with Th2 or mixed Th2/Th22 phenotypes are observed in allergic rhinitis and nasal polyposis [59].

Collectively, these observations indicate that OX40-OX40L axis is a central communication pathway linking epithelial activation to adaptive immune memory across barrier tissues. By amplifying OX40⁺ T-cell responses in the skin and upper and lower airways, this pathway disrupts epithelial homeostasis and sustains chronic inflammation, supporting the concept of type 2 diseases as tissue-specific manifestations of a shared pathogenic axis stabilized by immune persistence.

### OX40-OX40L as a Shared Axis of Type 2 Inflammation: A Pan-type 2 Perspective

Type 2 inflammation has evolved from a concept limited to classical allergic responses into an integrated framework encompassing multiple chronic diseases driven by shared immunological mechanisms. Core features of atopic dermatitis, asthma, allergic rhinitis, and nasal polyposis include Th2 cells, type 2 innate lymphoid cells, eosinophils, basophils, and the production of IL-4, IL-5, and IL-13, which collectively disrupt epithelial barrier integrity and perpetuate sensitization[60, 61].

Within this context, the OX40-OX40L axis emerges as a central immunological integrator linking distinct epithelial compartments and stabilizing type 2 immune responses across disease states [62]. OX40 signaling enhances Th2 cell survival, effector function, and tissue-resident memory (TRM) by facilitating the transition from acute adaptive responses to chronic and relapsing inflammation.

Consistent evidence across diseases supports this hypothesis. In atopic dermatitis, OX40-OX40L signaling sustains Th2 polarization and memory T cells in non-lesional skin [63]; in asthma, OX40 on T cells and OX40L on basophils and dendritic cells contribute to eosinophilic inflammation and airway hyperresponsiveness [64]; and in allergic rhinitis [65] and nasal polyposis [66, 67], OX40-OX40L expression correlates with disease severity and chronic upper airway inflammation.

Together, these observations support the concept of a shared type 2 inflammatory network, in which distinct clinical entities may represent tissue-specific manifestations of overlapping immune pathways involving OX40-OX40L signaling. This framework provides a strong biological rationale for therapeutic targeting of the OX40-OX40L pathway and for the development of shared biomarkers and intervention strategies aimed at restoring immune homeostasis across barrier tissues [68].

#### Atopic Dermatitis

**Fig. 2:**
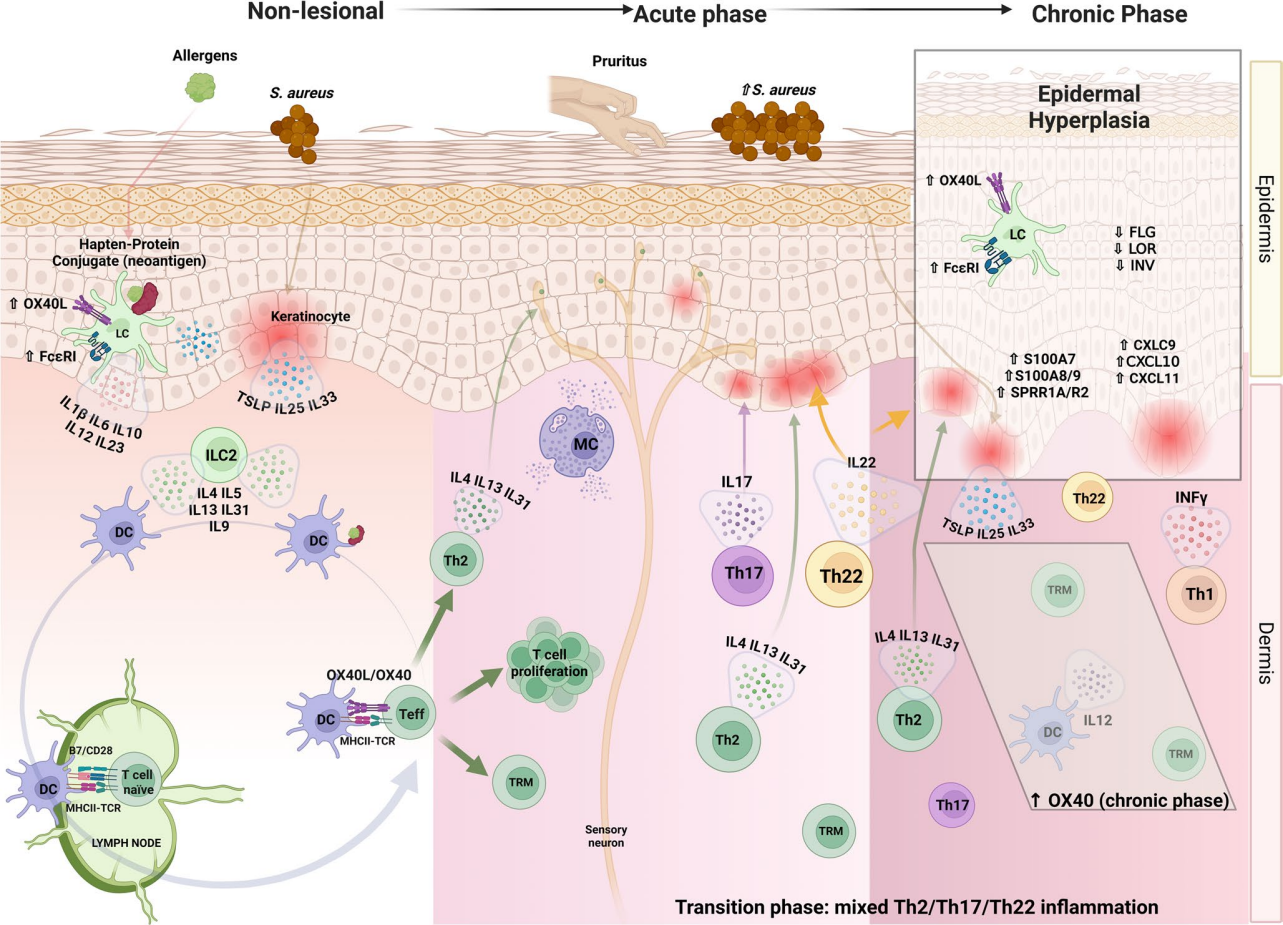
Temporal and spatial organization of the OX40-OX40L axis in atopic dermatitis. In atopic dermatitis, epidermal barrier dysfunction facilitates chronic exposure to allergens and microbial stimuli, including *Staphylococcus aureus*, leading to the release of epithelial alarmins by keratinocytes, such as TSLP, IL-25, and IL-33. These signals activate cutaneous antigen-presenting cells, including Langerhans cells and dermal dendritic cells, and initiate antigen-dependent T-cell priming in skin-draining lymph nodes through TCR-MHC recognition and early CD28-CD80/CD86 co-stimulation. Inducible co-stimulatory pathways of the TNFR superfamily, particularly the OX40-OX40L axis, become engaged within the skin following this initial activation phase. OX40 signaling promotes effector T cell expansion, survival, and functional persistence, reinforcing type 2 inflammation during acute disease and supporting a mixed Th2/Th17/Th22 immune profile during the transition to chronic inflammation. Sustained OX40-OX40L engagement contributes to the maintenance of skin-resident memory T cells, epidermal hyperplasia, and long-term inflammatory memory in atopic dermatitis, thereby facilitating disease chronicity and relapse. Created with BioRender.com

AD represents the paradigmatic form of type 2 skin inflammation and constitutes the most extensively studied human model of the OX40-OX40L axis. Epidermal barrier dysfunction and chronic exposure to allergens and microorganisms induce the release of epithelial alarmins, including TSLP, IL-33, and IL-25, by keratinocytes [69, 70]. These cytokines promote OX40L upregulation on dermal dendritic cells and support the expansion of OX40⁺ CD4⁺ T cells with a Th2 phenotype (Fig. 2). OX40-OX40L signaling sustains effector T-cell proliferation, differentiation, and survival while reducing suppressive regulatory T-cell function [2, 4, 71].

Activated T lymphocytes produce IL-4, IL-13, and IL-31, thereby worsening skin barrier dysfunction, increasing pruritus, and driving a self-perpetuating inflammatory cycle [72]. OX40 signaling promotes the generation and long-term maintenance of skin-resident memory T cells (TRM), which remain in clinically non-lesional skin and facilitate disease recurrence [73, 74]. In active AD lesions, immunohistochemical studies have demonstrated marked overexpression of OX40 on infiltrating lymphocytes and of OX40L on dendritic cells, mast cells, and dermal endothelial cells, with expression levels correlating with SCORAD scores and overall clinical severity [12, 75].

The role of this axis extends into the chronic phase of disease, where sustained OX40-OX40L signaling supports a mixed Th2/Th22 immune profile and is associated with the expansion of Th1 and Th17 subpopulations [62]. This suggests that OX40 may contribute to the transition from acute to chronic inflammation, supporting the survival of effector T cells and the establishment of long-term inflammatory memory. OX40 stimulation also activates anti-apoptotic mechanisms, including Bcl-2 and Bcl-xL signaling, thereby prolonging lymphocyte survival and contributing to inflammatory chronicity [2].

Inhibition of the OX40-OX40L pathway has emerged as a promising therapeutic strategy for managing moderate-to-severe AD. In phase 2 trials, monoclonal antibodies targeting this axis have demonstrated clinically meaningful improvements in disease severity, including reductions of approximately 50–60% in EASI scores, with generally favorable safety profiles [76, 77]. Beyond clinical efficacy, OX40-OX40L blockade may exert broader immunomodulatory effects by influencing Th1, Th17, and Th22 pathways in addition to type 2 inflammation. These findings suggest that OX40-OX40L-targeted therapies may induce broader immune modulation beyond transient cytokine suppression, although the long-term durability and disease-modifying implications of these responses remain under investigation [77].

#### Asthma

**Fig. 3:**
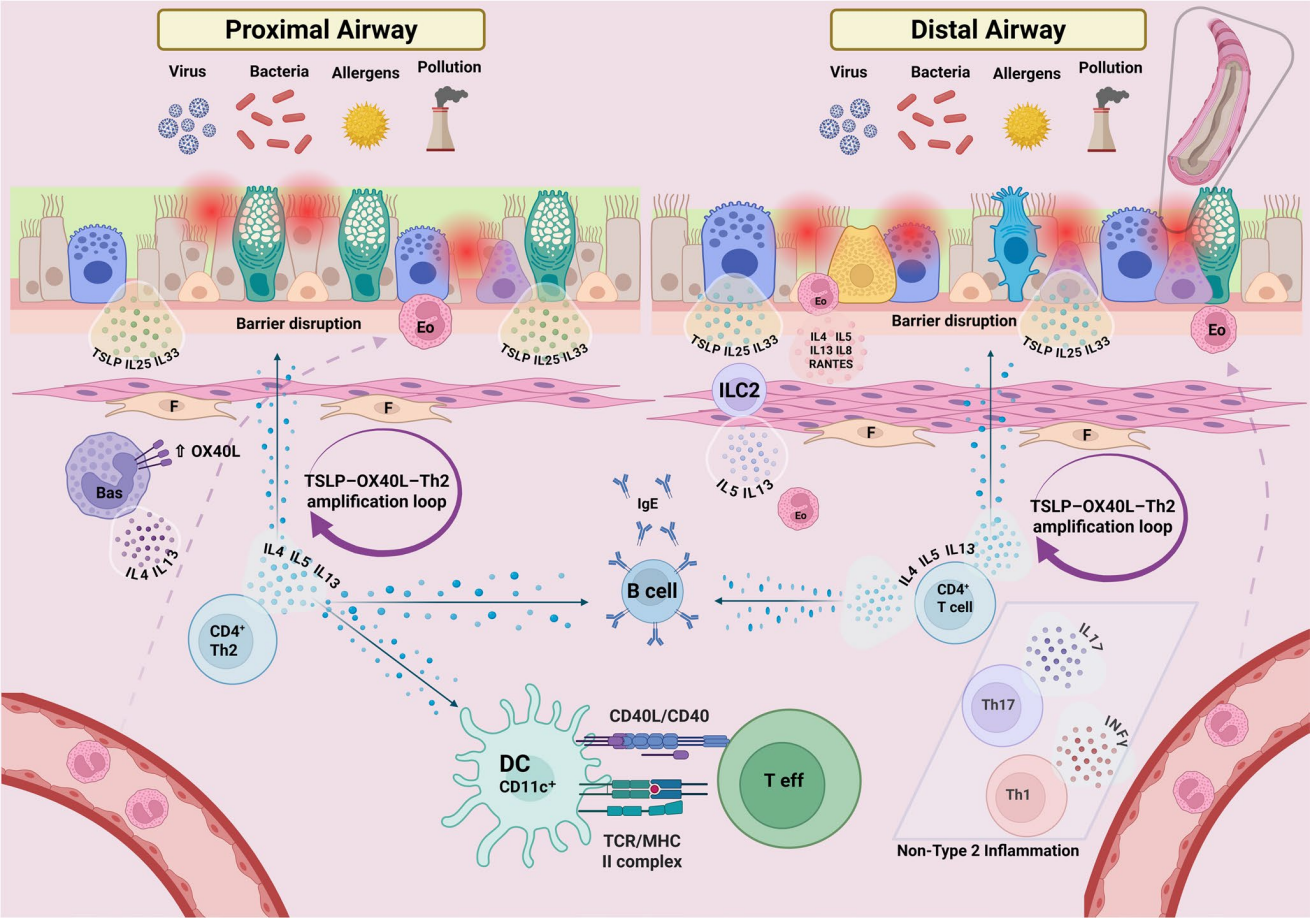
Spatial and immunological organization of the OX40-OX40L axis across the upper and lower airways in patients with allergic asthma. Environmental triggers, including allergens, viral and bacterial infections, and air pollution, induce the epithelial barrier throughout the upper and lower airways, releasing epithelial alarmins such as TSLP, IL-25, and IL-33. These signals activate CD11c⁺ dendritic cells and innate lymphoid cells type 2 (ILC2), promoting OX40L upregulation and the expansion of OX40-expressing CD4⁺ T cells following antigen-dependent priming. The engagement of the OX40-OX40L axis amplifies type 2 immune responses through a TSLP-OX40L-Th2 amplification loop, sustaining the production of IL-4, IL-5, and IL-13, eosinophilic inflammation, IgE class switching, mucus hypersecretion, and airway hyperresponsiveness. OX40 signaling supports the persistence of effector and memory T-cell populations and contributes to inflammatory heterogeneity, including the co-existence of Th17- and Th1-associated responses, particularly within distal airway compartments. Created with BioRender.com

The OX40-OX40L axis plays a central role in the amplification and persistence of type 2 inflammation in allergic asthma. Environmental triggers, including allergens, viral infections, and pollutants, promote epithelial alarmin release and OX40L upregulation within the bronchial mucosa, sustaining Th2-driven airway inflammation (Fig. 3) [9, 64].

The pathogenic relevance of OX40-OX40L signaling in asthma has been demonstrated across multiple experimental models [78, 79]. Genetic deficiency or antibody-mediated blockade of OX40 or OX40L reduces pulmonary eosinophilia, Th2 cytokine production, mucus secretion, and serum IgE levels in ovalbumin- or house dust mite-induced asthma murine models [80]. In addition, OX40 signaling supports the persistence and reactivation of effectors and memory CD4⁺ T cells within the lung, potentially contributing to disease chronicity and susceptibility to exacerbations.

Beyond dendritic cell-T cell interactions, basophils have been shown in selected experimental models to upregulate OX40L following allergen exposure and amplify key features of type 2 immune responses [58]. In human asthma, increased expression of OX40 and OX40L in the bronchial submucosa and bronchoalveolar lavage fluid correlates with eosinophilia [81] and local IL-4 expression, supporting the involvement of this pathway in type 2-driven airway inflammation [81]. Environmental stimuli such as diesel exhaust particles [82] and *Alternaria spp *[83] can further enhance OX40L expression in the respiratory epithelium.

In murine models of ovalbumin-induced asthma, recombinant OX40L administration or adoptive transfer of OX40⁺ T lymphocytes intensified eosinophilic inflammation and cytokine production, IL-4, IL-6, IL-13, IL-17, tumor necrosis factor-α, and interferon-γ [84], whereas OX40L neutralization reduced pulmonary inflammation, NF-κB activation, and T-cell proliferation [85]. Collectively, these findings support the existence of a TSLP-OX40L-Th2 inflammatory circuit to allergic airway inflammation and highlight the OX40-OX40L axis as a potential therapeutic target in asthma [86].

In models closely resembling human asthma, chronic exposure to house dust mite (*Dermatophagoides pteronyssinus*, HDM) has supported the role of the OX40-OX40L axis in allergic airway inflammation [79]. OX40 neutralization reduced inflammatory cell infiltration, effector and memory CD4⁺ T-cell responses, and IL-4, IL-5, IL-13, and IL-17 production, with parallel improvements in lung function and decreases in allergen-specific IgE and IgG1 [80]. These effects were maintained when treatment was initiated after disease establishment and were partially recapitulated in peripheral blood mononuclear cells from HDM-allergic individuals, supporting the translational relevance of these findings [87].

Notably, the OX40-OX40L axis has also been implicated in corticosteroid resistance in asthma by promoting the survival of Th2 effector and memory CD4⁺ T lymphocytes with reduced susceptibility to corticosteroid-induced apoptosis [88]. In pediatric patients, elevated serum OX40L levels are associated with steroid-resistant asthma, IgE and eosinophilia, as well as with poor lung function and asthma control, suggesting this pathway as both a therapeutic target and a potential biomarker in allergic asthma [89]. OX40L overexpression in atopic asthma correlates with atopy markers, including total serum IgE, supporting its involvement in disease pathophysiology [90].

#### Allergic Rhinitis and Conjunctivitis

Allergic rhinitis shares a Th2-dominated immunological profile [60]. In nasal mucosal biopsies from allergic patients, OX40 is upregulated on infiltrating CD4⁺ T cells, while OX40L is expressed on dendritic and endothelial cells. Circulating soluble OX40L levels correlate with disease severity, supporting its potential use as a surrogate marker of local inflammatory activity. Experimental studies have also shown that repeated allergen exposure enhances OX40L expression on nasal mucosa dendritic cells and promotes local type 2 inflammatory responses [91].

OX40L silencing reduces allergic symptoms, eosinophilic infiltration, and IL-5 production in ovalbumin-induced allergic rhinitis murine model while increasing regulatory T-cells, consistent with a shift toward a more tolerogenic immune profile [92]. In parallel, B lymphocytes from patients with allergic rhinitis express elevated levels of OX40L, correlating with total serum IgE concentrations and IL-4 production by CD4⁺ T cells [93]. In addition, innate lymphoid cells, particularly ILC2s, may contribute to upper airway inflammation through OX40-OX40L-dependent innate-adaptive immune interactions [94].

A comparable involvement of the OX40-OX40L axis has been described in allergic conjunctivitis. Increased OX40L expression in the conjunctival epithelium and resident dendritic cells correlates with the accumulation of OX40⁺ CD4⁺ T lymphocytes and with clinical disease severity [95]. OX40 or OX40L blockade attenuates eosinophilic infiltration and Th2 cytokine production while improving ocular inflammatory signs in experimental models [96].

Emerging clinical observations from patients treated with OX40-OX40L-targeting agents for atopic dermatitis or prurigo nodularis have also suggested possible improvement of associated allergic conjunctivitis symptoms. Although these findings remain preliminary and require formal evaluation, they are biologically consistent with the involvement of OX40-OX40L signaling across multiple type 2 inflammatory barrier tissues. This broader upstream immunomodulatory activity may differ mechanistically from cytokine-specific approaches, such as IL-4Rα or IL-13 inhibition, which primarily target downstream effector pathways.”

#### Nasal Polyposis

**Fig. 4:**
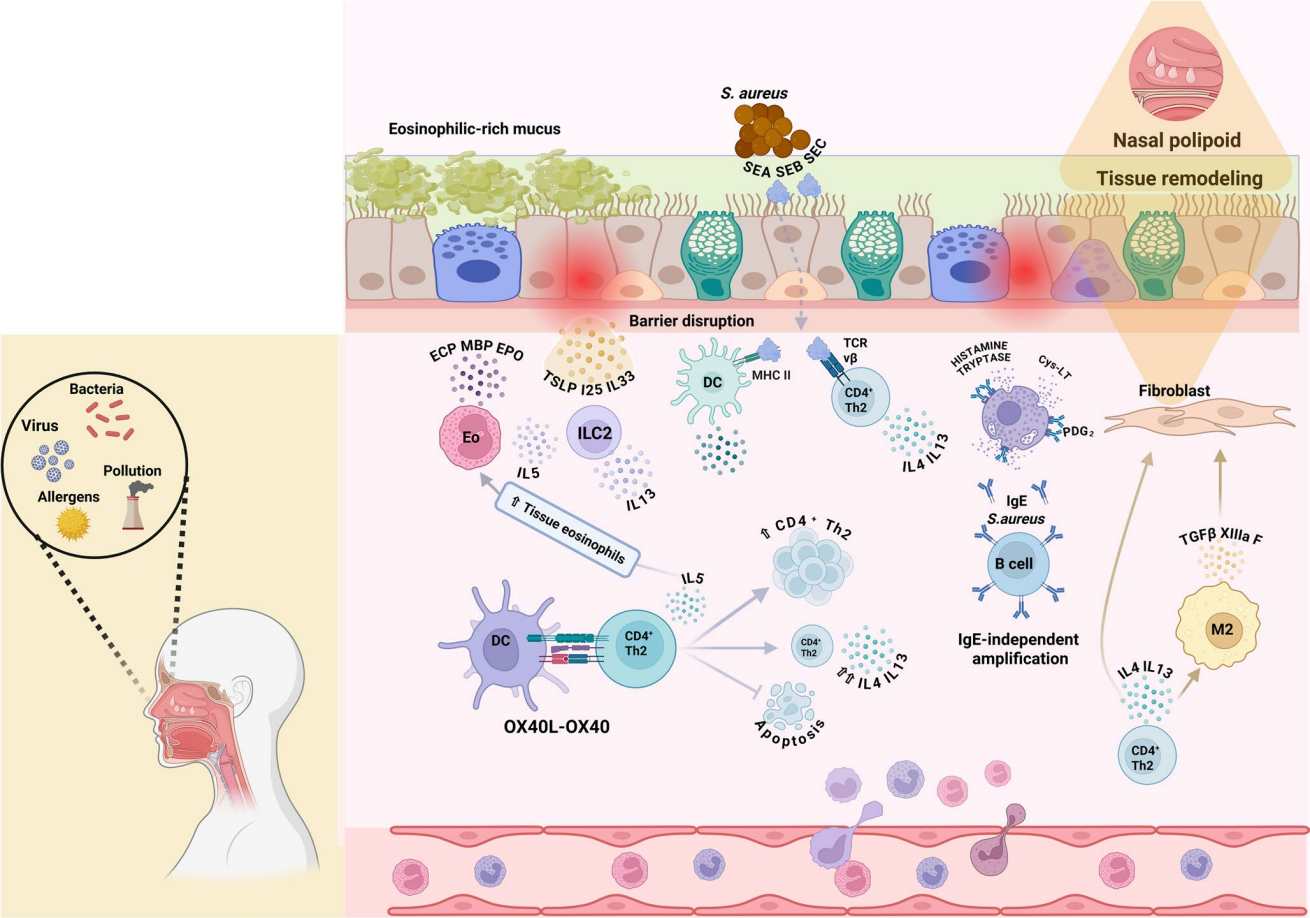
Immunological landscape and role of the OX40-OX40L axis in chronic rhinosinusitis with nasal polyps (CRSwNP). Persistent epithelial barrier dysfunction in the sinonasal mucosa, together with microbial stimuli, allergens, and environmental factors, induces the release of epithelial alarmins such as TSLP, IL-25, and IL-33. These signals promote activation of dendritic cells, innate lymphoid cells type 2 (ILC2), and other innate immune populations, leading to sustained type 2-skewed inflammation characterized by eosinophilic infiltration and cytokine production. Within the polyp tissue, OX40L is expressed by dendritic cells, eosinophils, mast cells, macrophages, and other stromal and immune cells, enabling bidirectional signaling with OX40-expressing CD4⁺ T cells. Engagement of the OX40-OX40L axis acts as a chronic co-stimulatory signal that supports effector T-cell survival and persistence, amplifies eosinophilic inflammation, and contributes to tissue remodeling through indirect activation of fibroblasts and type 2-associated myeloid pathways. Notably, this amplification loop may occur partially independently of classical IgE-mediated mechanisms and is further reinforced by microbial factors such as *Staphylococcus aureus*–derived superantigens. Collectively, these processes sustain chronic inflammation, promote polyp formation, and contribute to disease persistence and recurrence in CRSwNP. Created with BioRender.com

Persistent type 2 inflammation in chronic rhinosinusitis with nasal polyps (CRSwNP) is associated with frequent coexistence of asthma and atopy, prominent eosinophilic infiltration, and relative corticosteroid refractoriness [97]. This inflammatory endotype correlates with disease severity, recurrence, and comorbidities, and has been associated with increased OX40-OX40L signaling within the sinonasal mucosa (Fig. 4).

Clinical and tissue-level evidence support a pathogenic role of the OX40-OX40L axis in CRSwNP. Patients with nasal polyps display increased OX40L expression at both protein and mRNA levels [98], which is correlated with higher rates of atopy and asthma and tissue eosinophilia. OX40L was predominantly expressed within polyp tissue by dendritic cells, eosinophils, macrophages, mast cells, and CD3⁺ lymphocytes within the polyp stroma, supporting the involvement across innate and adaptive immune compartments.

OX40L overexpression occurs independently of allergic sensitization, suggesting an important contribution of epithelial and innate immune signaling beyond classical IgE-driven mechanisms [99]. These findings further support the involvement of the OX40-OX40L axis in the inflammatory microenvironment of CRSwNP.

#### Allergic Contact Dermatitis

**Fig. 5:**
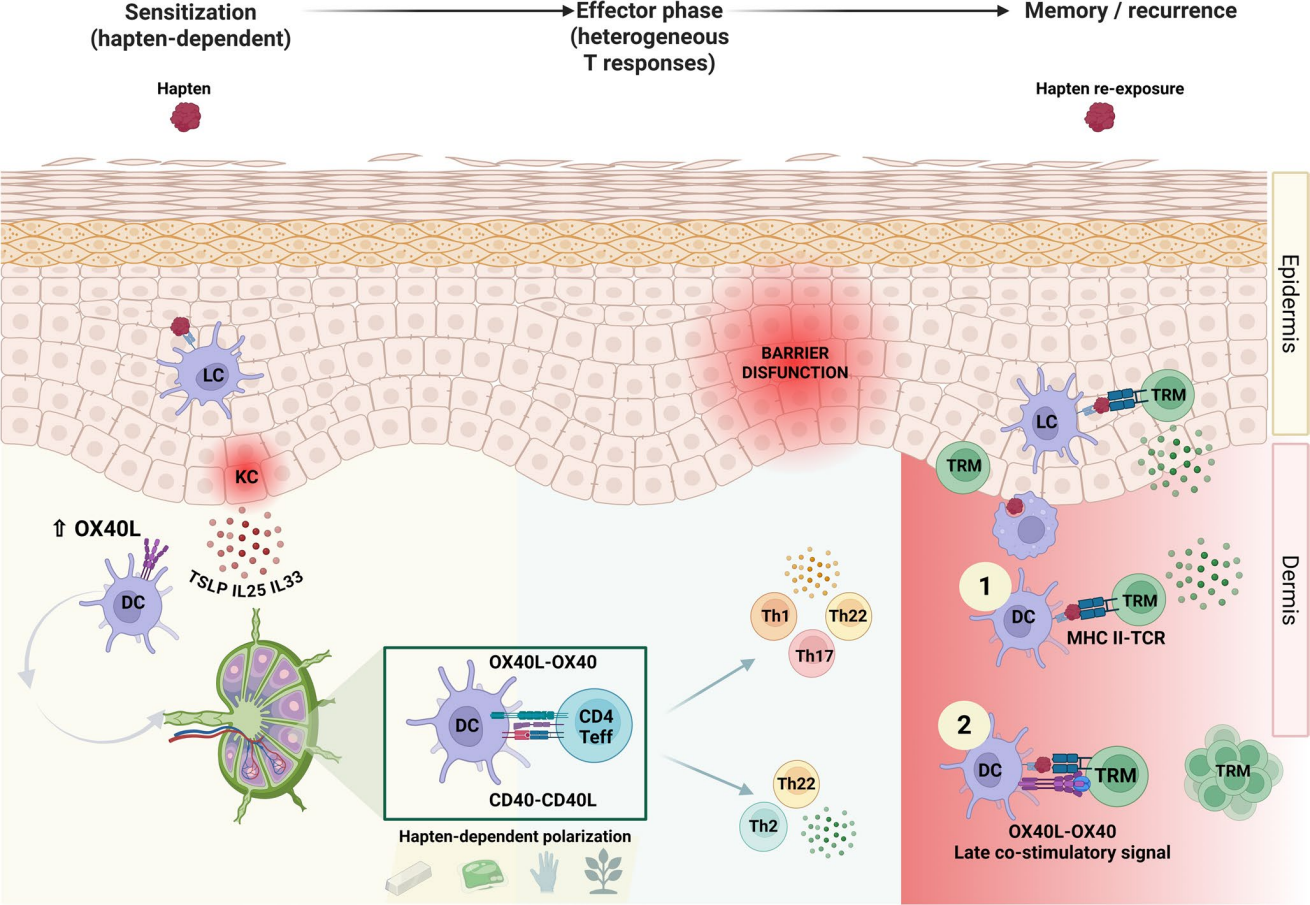
Temporal and immunological framework of allergic contact dermatitis, highlighting the OX40-OX40L axis’ role. Allergic contact dermatitis (ACD) develops through sequential and partially overlapping phases. During the sensitization phase, low-molecular-weight haptens penetrate the epidermis, bind to self-proteins, and are captured by Langerhans cells and dermal dendritic cells, which upregulate OX40L in response to epithelial-derived cytokines and migrate to draining lymph nodes to prime naïve T cells in a hapten-dependent manner. The effector phase is characterized by heterogeneous T-cell responses, with allergen-specific polarization toward Th1, Th17, Th22, and/or Th2 subsets depending on the nature of the hapten and the local cytokine milieu. Tissue-resident memory T cells (TRM) are rapidly reactivated locally upon hapten re-exposure through recognition of hapten-protein complexes presented by cutaneous antigen-presenting cells (MHC-TCR interaction), leading to cytokine release and barrier dysfunction. In this recall response, OX40-OX40L engagement functions as a late co-stimulatory signal that reinforces TRM survival, effector cytokine production, and local immune persistence, thereby contributing to disease chronicity and recurrence. Created with BioRender.com

Allergic contact dermatitis (ACD) represents a paradigmatic model of a type IV, T cell-mediated immune response triggered in the skin following exposure to low-molecular-weight haptens [100]. In this context, the OX40-OX40L axis contributes to both the sensitization and effector phases of contact hypersensitivity by promoting T-cell activation, expansion, and persistence (Fig. 5).

Experimental studies demonstrated that genetic or functional blockade of OX40L attenuates contact hypersensitivity responses, whereas OX40L overexpression intensifies cutaneous inflammation, lymphocyte proliferation, and cytokine production, including IL-2 and interferon-γ [101]. Following hapten exposure, epithelial and antigen-presenting cell activation promotes OX40L expression on Langerhans cells and dermal dendritic cells, supporting Th1-and Th17-skewed immune responses characteristic of contact hypersensitivity [102]. Accumulating evidence indicates that ACD is immunologically heterogeneous, but rather displays allergen-dependent inflammatory signatures. Despite similar clinical phenotypes, transcriptomic and molecular profiling studies have shown that distinct haptens elicit different patterns of T-cell polarization [103]. For example, nickel exposure is associated with prominent innate immune activation and predominant Th1/Th17 responses with a relevant Th22 component, whereas fragrance- and rubber-related allergens tend to induce comparatively greater Th2/Th22 skewing with weaker Th1/Th17 involvement [104].

In human ACD, OX40L expression has been detected on epidermal Langerhans cells, dermal dendritic cells, macrophages, and keratinocytes following allergen exposure, correlating with the presence of OX40⁺ CD4⁺ T lymphocytes and a mixed Th1/Th2/Th17 cytokine milieu [105]. OX40-OX40L blockade reduces resident memory T-cell proliferation and effector cytokine production, supporting the involvement of this pathway in local immune reactivation and chronic or recurrent disease [106].

#### OX40 Beyond type 2 Inflammation

The OX40-OX40L axis contributes to the chronic inflammatory network that sustains disease persistence. Increased frequencies of OX40⁺ CD4⁺ effector-memory T lymphocytes with a migratory, apoptosis-resistant phenotype have been identified in lesional skin, together with OX40L expression on myeloid and endothelial cells, supporting ongoing co-stimulatory signaling and chronic T-cell activation [107]. OX40 also regulates tissue-resident regulatory T cells (Tregs) in inflamed skin. Experimental data indicate that OX40 signaling may stabilize skin-resident Tregs by restraining Th17 differentiation, whereas loss of OX40 signaling promotes IL-17 A and RORγt expression, resulting in spontaneous cutaneous inflammation [108]. Table 2 summarizes the disease-specific immune contexts and functional consequences of OX40-OX40L signaling across inflammatory and immune-mediated dermatoses.

**Table 2:** Disease-specific roles of OX40-OX40L signaling

Disease	Immune context	Dominant T cells	OX40 role	Key outcome
AD	TSLP/IL-33	Th2/TRM	Memory persistence	Relapse
Asthma	DC2/basophils	Th2	Eosinophilia	Exacerbations
HS	TNF/Th17	Th17/Treg↓	Loss of regulation	Chronicity
CSU	Mast cell niche	Th2/Th17	IgE feedback	Refractoriness
Vitiligo	APC-driven	Th1/Th17	Cytotoxic memory	Progression
Pemphigus	Tfh–B	Tfh	Autoantibodies	Blistering
SSc/Morphea	Fibrotic	Th1	Fibroblast activation	Fibrosis
CTCL	Autocrine	Malignant T	Survival loop	Tumor growth

AD, atopic dermatitis; APC, antigen-presenting cell; CTCL, cutaneous T-cell lymphoma; CSU, chronic spontaneous urticaria; DC2, type 2 dendritic cell; HS, hidradenitis suppurativa; IgE, immunoglobulin E; SSc, systemic sclerosis; Tfh, T follicular helper cell; Th1, T helper 1 cell; Th2, T helper 2 cell; Th17, T helper 17 cell; TNF, tumor necrosis factor; Treg, regulatory T cell; TRM, tissue-resident memory T cell

### Autoimmune and Immune-dysregulated Dermatoses

Human studies in **hidradenitis suppurativa (HS)** have demonstrated dysregulation of TNFRSF-related pathways in regulatory T cells, with Th17 skewing and loss of regulatory control, highlighting the context-dependent role of OX40 in HS pathogenesis [109]. Transcriptomic analyses using tape-strip sampling have confirmed the upregulation of OX40 in both lesional and non-lesional HS skin, which correlates with disease severity and with TNF-α- and Th17-driven inflammatory signatures [110]. These findings support the concept of a subclinical inflammatory field and identify OX40 as a potential disease activity biomarker.

However, clinical evaluation of OX40 pathway inhibition as monotherapy did not demonstrate sufficient efficacy in HS, underscoring the limitations of single-target approaches in a disease characterized by marked immunological heterogeneity. Combined blockade of TNF-α and OX40L using the bispecific antibody SAR442970 (brivekimig) has demonstrated broader suppression of inflammatory gene programs than inhibition of either pathway alone, including reductions in T-cell proliferation and Th1, Th2, Th9, and Th17 signatures [111]. Currently under phase II clinical evaluation in HS, this strategy highlights the potential relevance of co-stimulatory pathway modulation in complex inflammatory dermatoses. Mast cells act as central orchestrators of immune activation in **chronic spontaneous urticaria (CSU)** within a complex dermal microenvironment enriched in T cells, macrophages, dendritic cells, and endothelial cells [112, 113]. While several studies support the involvement of type 2 cytokines and functionally relevant mast cell-T cell interactions in CSU, the disease is increasingly recognized as immunologically heterogeneous. Antihistamine-refractory CSU comprises distinct endotypes, including autoimmune type IIb disease, characterized by low total IgE levels and IgG-mediated mast cell activation rather than classical Th2 dominance. Accordingly, although a type 2-influenced microenvironment with enhanced mast cell-T cell crosstalk may characterize a subset of patients, current evidence supporting a uniformly M2-polarized macrophage niche in refractory CSU remains limited [114]. Constitutive and FcεRI-induced expression of OX40L on mast cells enables bidirectional signaling with OX40⁺ T cells, which may contribute to T-cell activation, effector persistence, and impaired regulatory control across different immunological contexts, including IgE-dependent and autoimmune-driven disease [115]. Collectively, these findings support the biological relevance of the OX40-OX40L axis in CSU, particularly within selected patient subsets, while underscoring the importance of endotype-informed therapeutic stratification.

In **vitiligo**, activation of the OX40-OX40L co-stimulatory axis has been associated with enhanced cytotoxic and helper T-cell responses, supporting a Th1/Th17-skewed immune profile and altered regulatory T-cell (Treg) function. Increased OX40-OX40L interactions between monocytes or antigen-presenting cells and CD4⁺ and CD8⁺ T cells potentiate the production of pro-inflammatory cytokines such as IFN-γ and TNF-α, driving melanocyte-directed immune responses and perpetuating cutaneous inflammation [116]. OX40 signaling may also impair Treg suppressive activity and support the persistence of autoreactive effector cells, contributing to immune dysregulation and disease chronicity. In addition, the involvement of this pathway in T follicular helper cell differentiation further supports a role of OX40-OX40L signaling in immune memory and relapse propensity [117]. Collectively, these findings support the involvement of the OX40-OX40L axis in vitiligo pathogenesis and maintenance.

Growing evidence supports the involvement of the OX40-OX40L axis in pemphigus pathogenesis. By promoting CD4⁺ T-cell differentiation toward a T follicular helper (Tfh) phenotype, OX40 signaling may enhance IL-21-dependent B-cell activation and contribute to the production of pathogenic anti-desmoglein autoantibodies48. Accordingly, inhibition of the OX40-OX40L pathway may reduce Tfh-mediated B-cell activation and autoantibody production, whereas persistent activation of this pathway could contribute to pathogenic humoral immune responses [118].

### Fibrotic Disorders

In keloids, increased OX40-OX40L expression together with reduced regulatory immune markers has been associated with sustained effector T-cell activation and chronic fibroinflammatory responses [119]. Keloid lesions are enriched in memory and tissue-resident T cells with reduced regulatory T-cell frequencies, suggesting a dysregulated immune microenvironment linked to chronic inflammation and recurrence [120]. Sustained OX40-OX40L signaling has also been associated with fibroblast activation, increased TGF-β1 production, and dermal remodeling [121]. OX40 expression in clinically non-lesional adjacent skin further suggests the presence of subclinical inflammatory activity that may contribute to disease persistence.

In sclerotic-type cutaneous chronic graft-versus-host disease, increased OX40L expression has been identified within fibroinflammatory immune signatures overlapping with systemic sclerosis [122]. In systemic sclerosis, OX40L is overexpressed in fibrotic skin and serum and correlates with inflammatory burden and disease progression, whereas experimental OX40L blockade attenuates inflammation-driven fibrosis in vivo [123]. Although morphea is classically Th1-skewed, OX40-OX40L signaling may similarly contribute to sustained immune activation and chronic fibroblast stimulation across the sclerodermiform spectrum [124].

### Lymphoproliferative Disorders

In **mycosis fungoides (MF) and Sezary syndrome (SS)**, neoplastic T cells aberrantly co-express both OX40 and its ligand OX40L, establishing an autocrine co-stimulatory loop that may promote malignant T-cell proliferation, survival, and resistance to apoptosis. This dual expression, which is absent in normal T lymphocytes, extends the relevance of OX40-OX40L signaling beyond inflammation to lymphoproliferative disorders. In a murine model, antibody-mediated blockade of OX40 or OX40L reduces the proliferation of cutaneous T-cell lymphoma (CTCL) cell lines, inhibits downstream AKT, ERK, p38, and JNK signaling pathways, and suppresses tumor growth [125].

Subsequent studies have supported the oncogenic relevance of OX40 signaling in CTCL. Papadavid et al. showed that OX40 genetic ablation in MyLa and SeAx CTCL cell lines reduced tumor growth, intravasation, and metastatic dissemination through ERK-dependent mechanisms [126]. OX40 expression promoted a Th2-skewed cytokine milieu, increased M2-polarized macrophage infiltration, and enhanced lymphangiogenesis via VEGF-C induction, contributing to a tumor-permissive microenvironment. Guglielmo et al. confirmed that coordinated OX40-OX40L activation in MF and SS engages NF-κB, PI3K/AKT, and BCL-2-dependent survival pathways, favoring clonal expansion and long-term persistence of malignant T cells, with expression levels correlating with clinical stage and tumor burden [127]. Together, these findings support the exploration of OX40-targeted strategies as potential therapeutic approaches in MF and SS.

## Discussion

According to experimental and clinical evidence, the OX40-OX40L axis is an important regulator of immune persistence at barrier tissues, contributing to the transition from transient immune activation to sustained type 2 inflammation [40]. Chronic activation of this pathway within inflamed epithelial environments may disrupt immune tolerance and support the survival and effector function of pathogenic T cells, contributing to disease chronicity and relapse across inflammatory disorders of the skin and mucosal surfaces [9].

OX40-OX40L signaling is subject to tissue-level regulation via ILC2-Treg feedback circuits. In IL-33-rich inflammatory niches, ILC2s may participate in local regulatory feedback interactions through OX40-OX40L-dependent crosstalk with tissue-adapted regulatory T cells [128]. Although ILC2s are predominantly recognized as amplifiers of type 2 inflammation and partners of Th2 responses, accumulating evidence highlights their context-dependent and bidirectional immunological functions [129]. Disruption of this regulatory balance may contribute to uncontrolled inflammation, underscoring the context-dependent nature of OX40-OX40L signaling in chronic type 2 immunity.

The functional outcome of OX40 signaling is highly dependent on cellular and spatial context. In non-type 2 settings, such as the tumor microenvironment, IL-33-activated ILC2s can enhance CD8⁺ T cell-mediated antitumor immunity through OX40-OX40L interactions [130], highlighting that this pathway is not intrinsically pro- or anti-inflammatory. However, sustained OX40-OX40L signaling converges into a shared pathogenic loop across barrier tissues in which recurrent immune activation is converted into stable tissue immune memory.

The frequent coexistence of atopic dermatitis, asthma, allergic rhinitis, and nasal polyposis may reflect anatomically distinct manifestations of shared immune memory circuits stabilized by OX40 signaling, rather than entirely independent diseases. This framework provides a biological explanation for clinical multimorbidity and supports the concept of pan-type 2 disease driven by overlapping pathogenic pathways. Importantly, whether early therapeutic modulation of the OX40-OX40L axis could modify the trajectory of atopic disease progression or influence the development of associated comorbidities remains an open and clinically relevant question that warrants prospective investigation.

Despite the broad pathogenic involvement of the OX40-OX40L axis across multiple inflammatory and immune-mediated diseases, current therapeutic development remains predominantly focused on atopic dermatitis, where mechanistic, translational, and phase II/III clinical evidence is strongest. In other conditions, including asthma, chronic rhinosinusitis with nasal polyps, allergic contact dermatitis, fibrosing disorders, and cutaneous T-cell lymphomas, available evidence supporting OX40-OX40L-directed therapies remains largely preclinical or early translational. Therefore, the broader therapeutic applicability of this pathway across non-AD diseases still requires formal clinical validation.

Clinical observations in AD support this integrative framework, with OX40 overexpression on skin-homing memory CD4⁺ T cells, colocalization with OX40L-expressing mast cells, and systemic alterations in soluble OX40 levels correlating with disease severity [12]. In addition to amplifying effector responses, sustained OX40-OX40L signaling disrupts local immune tolerance by impairing regulatory T-cell differentiation and suppressive function, thereby favoring persistent immune activation and durable tissue-specific immune memory.

Furthermore, OX40-driven immune memory exhibits marked functional plasticity, extending beyond Th2 polarization toward Th17 and Th22 programs. The co-expression of OX40 with IL-22 and IL-17 A has been linked to deeper barrier disruption and more persistent disease in AD [13], whereas sustained OX40-OX40L signaling contributes to chronic immune activation and disease persistence in ACD and cutaneous T-cell lymphomas [131]. Collectively, these findings support the involvement of OX40-OX40L signaling across multiple inflammatory and lymphoproliferative skin disorders. Despite the encouraging clinical and mechanistic rationale supporting OX40-OX40L targeting, several limitations remain. Long-term safety and durability data beyond approximately 3–5 years are still limited, particularly regarding sustained disease control, relapse patterns after repeated treatment cycles, and potential class-specific risks. OX40 signaling is physiologically involved in the generation and maintenance of protective immune memory; the long-term immunological consequences of chronic or repeated OX40 modulation, including potential effects on host defense, vaccine responses, and immune surveillance, remain poorly understood. Given the physiological role of OX40 signaling in adaptive immune memory, long-term modulation of this pathway may theoretically affect immune surveillance, vaccine responses, and host defense mechanisms. Moreover, recent safety observations, including malignancy-related signals reported with some OX40-targeting approaches, underscore the need for long-term pharmacovigilance and careful mechanistic differentiation between receptor-depleting and ligand-directed strategies. Further longitudinal studies, real-world registries, and biomarker-driven analyses are required to better define the long-term immunological effects, durability, and safety of OX40-directed therapies without compromising protective immune functions.

An additional layer of complexity arises from the immune system’s intrinsic redundancy, which is structured around multiple overlapping co-stimulatory pathways and regulatory “checks and balances.” In this context, alternative TNFR family members or parallel co-stimulatory signals, such as OX40-OX40L, may partially compensate for a single immune axis’s modulation, potentially limiting therapeutic efficacy in some disease settings or patient subsets. At the same time, this redundancy may confer a degree of safety by preserving essential immune functions despite targeted intervention. Recognizing this balance between robustness and adaptability is critical for interpreting variable clinical responses and guiding the rational development of combination strategies or biomarker-driven patient stratification in future OX40-based therapeutic approaches.

Finally, the OX40-OX40L axis operates within a broader, partially redundant co-stimulatory network. Overlapping roles between OX40L and CD30L have been demonstrated in IL-1- and IL-22-producing T cells, where CD30L blockade recapitulates the anti-inflammatory effects of OX40L inhibition in experimental models. OX40-OX40L targeting may not only suppress inflammation but also modulate local immune circuits involved in epithelial homeostasis and immune persistence at barrier tissues.

### Translational and Therapeutic Perspectives

Conventional immunosuppressive therapies broadly inhibit T-cell activity and may affect protective immune functions [102]. In contrast, targeting the OX40-OX40L axis represents a more selective immunomodulatory strategy, as OX40 expression is largely restricted to activated T cells. Preclinical studies have shown that OX40-OX40L blockade can attenuate effector T-cell-driven inflammation while preserving antiviral and innate immune responses [132]. Early clinical experience summarized in Table 3 further supports the therapeutic potential of OX40-OX40L-directed therapies across chronic inflammatory diseases.

**Table 3:** OX40/OX40L-targeted therapies are currently under clinical development

Drug	Target	Mechanism of action	Disease	Trial phase/status	Primary reported outcomes/observations
Rocatinlimab	OX40	Depleting IgG1 monoclonal antibody	AD	Phase III/development discontinued	Significant EASI improvement and sustained responses observed in phase III trials; following emerging safety observations currently under further evaluation (ir.kyowakirin.com)
Amlitelimab	OX40L	Non-depleting IgG4 monoclonal antibody	AD	Phase III	Reduction in EASI scores and improvement in disease activity
Telazorlimab	OX40	Blocking monoclonal antibody	AD	Phase II	Improvement in clinical disease severity during treatment
SAR442970 (brivekimig)	TNF-α + OX40L	Bispecific monoclonal antibody	HS	Phase II	Reduction in inflammatory gene signatures and T-cell activation

Within the evolving therapeutic landscape of chronic inflammatory dermatoses and airway diseases, OX40-OX40L-targeted therapies should be interpreted in the context of already established immune-directed approaches. IL-4Rα blockade and selective IL-13 inhibition have demonstrated robust efficacy across multiple type 2 inflammatory diseases, whereas JAK inhibitors provide broader and often rapid suppression of inflammatory signaling pathways. In comparison, OX40-OX40L modulation represents an upstream co-stimulatory approach with the potential to influence effector and memory T-cell responses across multiple immune axes. However, the long-term durability, comparative efficacy, safety profile, and potential disease-modifying implications of these therapies remain under active investigation and require confirmation in larger clinical studies.

### Rocatinlimab

A clinical proof of concept for targeting the OX40 pathway has been established in atopic dermatitis with rocatinlimab (KHK4083/AMG 451), a non-fucosylated IgG1 monoclonal antibody with selective depleting activity against activated OX40⁺ effector and memory T cells. By targeting a late co-stimulatory receptor preferentially expressed on pathogenic T cells, rocatinlimab exemplifies a receptor-directed strategy aimed at immune rebalancing rather than transient suppression of downstream inflammatory pathways.

In randomized, placebo-controlled clinical trials in adults with moderate-to-severe AD, rocatinlimab induced robust and dose-dependent clinical improvements that increased progressively during treatment and were sustained for several months after treatment discontinuation, supporting a disease-modifying effect rather than simple pharmacological carryover [133, 134]. These benefits extended across objective disease severity measures and patient-reported outcomes, including pruritus and quality of life.

The clinical activity of rocatinlimab has also been supported by phase 3 studies demonstrating consistent efficacy across validated clinical endpoints and patient subgroups, with an overall safety profile generally comparable to other biological therapies [135]. However, recent safety evaluations have raised concerns regarding malignancies potentially related to OX40 pathway modulation, including reported cases of Kaposi sarcoma, which have led to the discontinuation of ongoing clinical studies. The available safety data are being further assessed.

### Amlitelimab

Amlitelimab (KY1005/SAR445229) is a fully human, non-depleting IgG4 monoclonal antibody that targets the OX40 ligand on antigen-presenting cells. Clinical efficacy and durability have been demonstrated in adults with moderate-to-severe AD in phase II randomized, placebo-controlled studies, including the STREAM-AD program [136]. Amlitelimab treatment resulted in rapid, dose-dependent improvements in disease severity that were sustained during extended follow-up.

Notably, a distinctive feature of amlitelimab is the persistence of clinical benefit after treatment discontinuation. In STREAM-AD, a substantial proportion of responders maintained clinical improvement for several months after drug withdrawal, despite declining serum concentrations, supporting a true disease-modifying effect rather than residual pharmacological activity [137, 138].

Amlitelimab was generally well tolerated, with adverse event rates comparable to those of placebo and no signal for hypersensitivity or serious infections, consistent with its non-depleting mechanism.

The clinical efficacy of amlitelimab has also been demonstrated by three different phase III studies that showed consistent efficacy across all clinical endpoints and patient subgroups. with a good safety profile, comparable to other biological therapies [139]. In contrast, Amlitelimab has not shown evidence of this safety signal in the data reported to date. Its safety profile, therefore, appears more favorable, which may be attributable to its non-depleting mechanism of action. By modulating immune responses without directly depleting target cell populations, Amlitelimab may preserve immune surveillance functions, potentially reducing the risk of malignancy observed with other approaches targeting the same pathway.

Collectively, these findings position OX40L blockade with amlitelimab as a mechanistically distinct and durable therapeutic strategy in atopic dermatitis, with the potential to enable extended dosing intervals and reduced treatment burden during ongoing phase 3 development.

### Telazorlimab

Telazorlimab (previously ISB 830 or GBR 830) is a humanized monoclonal antibody that targets the OX40 receptor on activated effector and memory T cells, modulating OX40-mediated co-stimulatory signaling with only moderate depleting activity. In contrast to ligand-directed or strongly depleting strategies, telazorlimab primarily inhibits receptor signaling on OX40⁺ T cells.

Clinical efficacy has been demonstrated in adults with moderate-to-severe AD in randomized, placebo-controlled phase 2 studies, with clinically meaningful improvements in disease severity observed during active treatment and maintained throughout extended on-treatment follow-up [140]. Telazorlimab was generally well tolerated, with an adverse event profile comparable to that of placebo, and no major safety signals were reported.

However, in contrast to OX40L-blocking or depleting approaches, durable off-treatment disease control has not yet been consistently demonstrated, and phase 3 data remain limited. At present, no ongoing clinical trials are evaluating telazorlimab in AD. Instead, a next-generation monoclonal antibody, STAR-0310, has been developed based on telazorlimab to improve its pharmacological and therapeutic properties.

### Future Perspectives

Therapies targeting the OX40-OX40L axis represent a conceptual shift in AD management, moving beyond chronic inflammatory suppression toward durable immune recalibration. These approaches offer the potential for long-term disease control and, in selected patients, sustained remission by selectively interfering with pathogenic T-cell survival and memory [63, 76, 141]. Advances in antibody engineering, including extended half-life formulations, may further enhance clinical feasibility by enabling less frequent dosing and improved adherence. However, the long-term effects of sustained OX40-OX40L modulation on regulatory T cells and tissue-resident memory compartments remain poorly understood and warrant careful longitudinal evaluation.

Distinct mechanistic profiles are beginning to emerge within this therapeutic class, reflecting different modes of ligand- versus receptor-directed modulation. These strategies appear to differ in the depth and durability of immune effects [63, 137, 142, 143], suggesting that the clinical value of OX40-OX40L targeting may lie less in short-term efficacy gains over existing biologics than in its capacity to support treatment de-escalation, induction–maintenance paradigms, and reduced long-term treatment burden if durable off-treatment responses are consistently confirmed [144].

Future integration of OX40-OX40L-directed therapies into atopic dermatitis management will require optimized patient selection, immune recalibration predictive biomarkers, and rational sequencing with established biologics and JAK inhibitors. This axis offers a mechanistically distinct approach with the potential to redefine long-term treatment goals in a disease traditionally considered lifelong and relapsing by targeting pathogenic T-cell persistence rather than downstream cytokine signaling [145]. In this context, positioning OX40-OX40L as a central regulator of immune persistence opens the door to future integrated or combinatorial strategies that coordinate multiple immune pathways, with OX40 signaling acting as a key anchoring axis for durable immune recalibration.

## Conclusion

The OX40-OX40L axis emerges as a central regulator of immune persistence at barrier tissues, linking epithelial danger signals to long-lived adaptive immune memory. By sustaining effector and tissue-resident T-cell programs while impairing regulatory control, this pathway contributes not only to type 2 inflammation but also to autoimmune, fibrosing, and lymphoproliferative skin diseases. Rather than acting as a simple amplifier of inflammation, OX40-OX40L signaling orchestrates a pathological immune memory system that converts recurrent tissue stress into stable disease states, providing a unifying framework for chronicity, relapse, and multimorbidity across skin and mucosal inflammatory disorders. From a therapeutic perspective, targeting this axis offers the opportunity to reprogram immune persistence rather than merely suppressing downstream mediators, redefining long-term disease control in chronic barrier inflammation.

## Data Availability

No datasets were generated or analysed during the current study.
