# Correction: The OX40-OX40L Co-Stimulatory Pathway as a Shared Driver of Immune Persistence in Skin and Airway Inflammation

**DOI:** 10.1007/s12016-026-09188-w

**Published:** 2026-08-04

**Authors:** Francisco José Navarro-Triviño, Tiago Torres, Ricardo Ruiz-Villaverde, José Luis Moreno-Amador, Pedro Mendes-Bastos

**Affiliations:** 1 Universidad de Granada, Granada, Spain; 2 Servicio de Dermatología, Hospital Universitario San Cecilio, Granada, Spain; 3 Instituto Biosanitario de Granada (ibs.GRANADA), Granada, España; 4 Serviço de Dermatologia, Centro Hospitalar Universitário do Porto, Instituto de Ciências Biomédicas Abel Salazar, Universidade do Porto, Porto, Portugal; 5 Sanofi, Madrid, Spain; 6 Serviço de Dermatologia, Hospital CUF Descobertas, Lisboa, Portugal

**Correction: Clinical Reviews in Allergy & Immunology (2026) 69:59**

10.1007/s12016-026-09187-x

The original online version of this article has been revised. Part of the caption for Figure 2:

“In atopic dermatitis, epidermal barrier dysfunction facilitates chronic exposure to allergens and microbial stimuli, including *Staphylococcus aureus*, leading to the release of epithelial alarmins by keratinocytes, such as TSLP, IL-25, and IL-33. These signals activate cutaneous antigen-presenting cells, including Langerhans cells and dermal dendritic cells, and initiate antigen-dependent T-cell priming in skin-draining lymph nodes through TCR-MHC recognition and early CD28-CD80/CD86 co-stimulation. Inducible co-stimulatory pathways of the TNFR superfamily, particularly the OX40-OX40L axis, become engaged within the skin following this initial activation phase. OX40 signaling promotes effector T cell expansion, survival, and functional persistence, reinforcing type 2 inflammation during acute disease and supporting a mixed Th2/Th17/Th22 immune profile during the transition to chronic inflammation. Sustained OX40-OX40L engagement contributes to the maintenance of skin-resident memory T cells, epidermal hyperplasia, and long-term inflammatory memory in atopic dermatitis, thereby facilitating disease chronicity and relapse. Created with BioRender.com”

was inadvertently included as a regular paragraph under the “Atopic Dermatitis” section.

The original article has been corrected.

